# Molecular Engineering of a Fluorescent Bioprobe for Sensitive and Selective Detection of Amphibole Asbestos

**DOI:** 10.1371/journal.pone.0076231

**Published:** 2013-09-27

**Authors:** Takenori Ishida, Maxym Alexandrov, Tomoki Nishimura, Ryuichi Hirota, Takeshi Ikeda, Akio Kuroda

**Affiliations:** Department of Molecular Biotechnology, Higashihiroshima, Hiroshima, Japan; University of Crete, Greece

## Abstract

Fluorescence microscopy-based affinity assay could enable highly sensitive and selective detection of airborne asbestos, an inorganic environmental pollutant that can cause mesothelioma and lung cancer. We have selected an *Escherichia coli* histone-like nucleoid structuring protein, H-NS, as a promising candidate for an amphibole asbestos bioprobe. H-NS has high affinity to amphibole asbestos, but also binds to an increasingly common asbestos substitute, wollastonite. To develop a highly specific Bioprobe for amphibole asbestos, we first identified a specific but low-affinity amosite-binding sequence by slicing H-NS into several fragments. Second, we constructed a streptavidin tetramer complex displaying four amosite-binding fragments, resulting in the 250-fold increase in the probe affinity as compared to the single fragment. The tetramer probe had sufficient affinity and specificity for detecting all the five types of asbestos in the amphibole group, and could be used to distinguish them from wollastonite. In order to clarify the binding mechanism and identify the amino acid residues contributing to the probe’s affinity to amosite fibers, we constructed a number of shorter and substituted peptides. We found that the probable binding mechanism is electrostatic interaction, with positively charged side chains of lysine residues being primarily responsible for the probe’s affinity to asbestos.

## Introduction

Interaction between proteins or peptides and inorganic materials is an important research subject in various fields of applied research, from nanotechnology to the development of bioassays, biosensors and biocompatible materials [1]. One of the emerging applications for the material-binding proteins and peptides is the fluorescence microscopy-based affinity assay for asbestos, a common inorganic pollutant that can cause asbestosis, mesothelioma and lung cancer [2-4]. Asbestos is a set of six fibrous silicate minerals that have been widely used in various construction materials because of their chemical and thermal stability [5]. Although the use of asbestos is now prohibited in most developed countries, large amounts of asbestos still remain in many older buildings. Airborne asbestos fibers could be generated when the asbestos-containing construction materials are damaged, which commonly happens during renovation or demolition work. Asbestos contamination therefore remains a major problem, with many countries reporting rising incidence of asbestos-linked pleural mesothelioma and lung cancers [6,7].

Asbestos is generally classified into two mineral groups, which differ in their crystal structure and toxicity [5]. Chrysotile (Mg_6_Si_4_O_10_(OH)_8_), a member of serpentine mineral group, accounts for more than 90% of industrially used asbestos [5]. We have already identified a highly specific protein probe for chrysotile by screening bacterial lysate for chrysotile-binding proteins [2,3]. The objective of this research is to develop a probe that selectively binds to asbestos fibers in the amphibole mineral group, which include amosite ((Fe, Mg)_7_Si_8_O_22_(OH)_2_) and crocidolite (Na_2_(Fe^3+^)_2_(Fe^2+^)_3_Si_8_O_22_(OH)_2_), as well as tremolite, actinolite, and anthophyllite. These five types of amphibole asbestos have very similar crystal structure and surface properties [5], making it possible to use amosite asbestos for screening and development of a cross-reactive amphibole asbestos probe.

Sensitive and selective detection of asbestos fibers requires a probe that has (a) sufficient binding affinity to visualize extremely thin asbestos fibers under fluorescence microscope, and (b) high specificity to asbestos to avoid staining of non-asbestos fibers, in particular those widely used in construction industry. However, previously identified amphibole asbestos-binding proteins from bacterial lysate were found to also bind to the natural fibrous mineral wollastonite (CaSiO_3_), an increasingly common asbestos substitute. A possible explanation for the lack of specificity is the presence of multiple binding sequences with different affinities and specificities. Therefore, in order to develop a more selective probe for amphibole asbestos, we attempted to isolate a protein fragment that is specific for amosite. One of the promising candidates for the amphibole asbestos-binding probe, *Escherichia coli* histone-like nucleoid-structuring (H-NS) protein, was sliced into several fragments, leading to identification of a specific but low-affinity amosite binding sequence. To increase the affinity of the probe, we constructed a streptavidin-based tetramer displaying four H-NS fragments with the amosite binding sequence. As expected, the engineered probe had high affinity to amosite and other types of asbestos in the amphibole group, combined with sufficient specificity to distinguish asbestos from wollastonite.

## Materials and Methods

### Materials

Amosite (JAWE 231) asbestos was obtained from Japan Association for Working Environment Measurement (Tokyo, Japan). Wollastonite was provided by the Japan Fibrous Material Research Association [8]. Plasmid pET21-b was purchased from Novagen (Merck KGaA, Darmstadt, Germany). Cy3 Maleimide mono-Reactive dye was purchased from GE Healthcare (Chalfont St. Giles, UK). Streptavidin-Cy3 was purchased form Invitrogen (Carlsbad, CA). Biotinylated peptides were purchased from Operon Biotechnologies (Tokyo, Japan). All other reagents were purchased from Wako Chemicals (Tokyo, Japan) or Sigma (St. Louis, MO) and were of the highest available quality.

### Identification of Amphibole Asbestos-Binding Proteins

*E. coli* cells grown overnight in 2xYT medium were inoculated into LB medium, incubated at 28 °C for 6 h, collected by centrifugation, and disrupted in the presence of 0.25 mg/ml of lysozyme by ultrasonication (Branson, CT). The lysate was centrifuged at 20,000*g* for 15 min. The protein concentration of the cleared supernatant was adjusted to 1 mg/ml with 25 mM Tris-HCl buffer (pH 7.5) containing 0.5% Tween 20. In order to eliminate proteins that have affinity to rock wool, which is widely used in construction industry as a safer substitute for asbestos, rock wool (50 mg) was added to 10 ml of the diluted supernatant, incubated for 1 h at 4 °C with rotary mixing, and precipitated by centrifugation at 10,000*g* for 10 min. The supernatant was filtrated to remove rock wool completely. Amosite asbestos (5 mg) was added to 10 ml of the filtrated supernatant, incubated for 10 min at room temperature with rotary mixing and precipitated by centrifugation at 10,000*g* for 10 min. The precipitated fibers were washed three times with 1 ml of wash buffer containing 25 mM Tris-HCl (pH7.5), 0.5 M NaCl and 0.5% Tween 20. Proteins bound to fibers were eluted by boiling for 5 min in 100 µl of SDS-sample buffer containing 100 mM Tris-HCl (pH6.8), 2% SDS, 8% glycerol, 4% 2-mercaptoethanol, and 0.05% bromophenol blue, and subsequently separated by 12.5% SDS-polyacrylamide gel electrophoresis. To perform mass spectrometric analysis, protein bands were excised from Coomassie blue-stained gels and in-gel digestion of the proteins was performed using sequencing-grade trypsin (Promega, Madison, WI). For matrix-assisted laser desorption/ionization-time of flight (MALDI-TOF) analysis, the peptide extracts were directly applied onto the MALDI target and analyzed with a MALDI-TOF apparatus (Bruker, Bremen, Germany). The peptide fingerprints obtained by MALDI-TOF were used for protein searches by Mascot (Matrix science Ltd, London, UK).

### Construction of Plasmids

Primers used in this study are listed in Table **1**, and *h*-*ns* gene fragments cloned into the plasmids are listed in File **S1**. The structural gene for *h-ns* (accession number in GenBank: AAC74319) was amplified by polymerase chain reaction (PCR) using the primer sets P1/P2 and *E. coli* MG1655 DNA as a template. The amplified h-ns gene was inserted into the *Nde*I and *Bam*HI sites of pET21-b (Novagen/Merck KGaA, Darmstadt, Germany). The resulting plasmid was designated pET-HNS. To generate plasmids for expressing H-NS protein fragments H-NS_1-59_, H-NS_60-137_, and H-NS_60-90_, the corresponding DNA sequences were amplified with primer sets P1/P3, P4/P2, and P4/P5, respectively, and then inserted into the *Nde*I and *Bam*HI sites of pET21-b. The resulting plasmids were designated pET-HNS_1-59_, pET-HNS_60-137_, and pET-HNS_60-90_. To generate plasmids for H-NS protein fragments connected to a 15-amino acid biotin-acceptor peptide (AviTag), we first constructed a plasmid expressing this peptide by inverse PCR using primers P7 and P8 with pET21-b as template. In the resulting plasmid, designated pET-AviTag, AviTag sequence was inserted into the flanking region of HisTag. Subsequently, DNA sequences encoding H-NS_60-137_, H-NS_60-90_, and H-NS_91-137_ fragments were amplified with primer sets P4/P2, P4/P5 and P6/P2, respectively, and then inserted into the *Nde*I and *Bam*HI sites of pET-AviTag to generate pET-HNS_60-137_-AviTag, pET-HNS_60-90_-AviTag, and pET-HNS_91-137_-AviTag.

**Table 1:** Sequences of primers.

**Primer**	**DNA sequence**
P1	GAATTCCATATGATGAGCGAAGCACTTAAAATTC
P2	GGATCCAAACATTGCTTGATCAGGAAATCG
P3	GGAGGATCCAAACACTGCAGTTTACGAGTGCGC
P4	CATATGCAATATCGCGAAATGCTGATC
P5	GGATCCAAACAACGTTTAGCTTTGGTGCC
P6	CATATGGCTCAGCGTCCGGCAAAATATAG
P7	GCTCAGAAAATCGAATGGCACGAACACCACCACCACCACCACTGAACTA
P8	CTCGAAGATGTCGTTCAGACCGCCACCCTCGAGTGCGGCCGCAAGCTTGTC

### Expression and Purification of the Recombinant Proteins

Plasmids (pET-HNS, pET-HNS_1-59_, pET-HNS_60-137_, and pET-HNS_60-90_) were introduced into *E. coli* Rosetta (DE3) pLysS (Novagen/Merck KGaA, Darmstadt, Germany). Cells were grown at 37 °C in LB medium supplemented with ampicillin (100 µg/ml) and chloramphenicol (30 µg/ml). Once the optical density of the cells at 600 nm reached 0.5, 0.5 mM isopropyl-β-d-thiogalactopyranoside (IPTG) was added to the medium. After another 4 h of cultivation at 28 °C, cells were harvested by centrifugation, and the pellet was stored at -80 °C until use. Plasmids expressing protein fragments fused to the AviTag (pET-HNS_60-137_-AviTag, pET-HNS_60-90_-AviTag, and pET-HNS_91-137_-AviTag) were introduced into *E. coli* BL21(DE3) (Novagen/Merck KGaA, Darmstadt, Germany) harboring pBirAcm (Avidity, LLC, Aurora, CO). Cells were grown at 37 °C in TYH medium [20 g/l tryptone, 10 g/l yeast extract, 11 g/l HEPES (pH7.2), 5 g/l NaCl, 1 g/l MgSO_4_] supplemented with 0.5% (w/v) glucose, 0.1 mM D-biotin, ampicillin (100 µg/ml), and chloramphenicol (30 µg/ml). Once the optical density of the cells at 600 nm reached 0.5, IPTG (0.5 mM) was added to the medium. After another 4 h of cultivation at 28 °C, cells were harvested by centrifugation, and the pellet was stored at -80 °C until use. The recombinant *E. coli* cells producing H-NS were suspended in 10 ml of 50 mM Tris-HCl buffer (pH8.3) containing 50 mM NaCl and 10% glycerol, and disrupted with a Digital Sonifier 450. The lysate was centrifuged at 100,000*g* for 20 min. Supernatant containing H-NS protein with C-terminal HisTag was purified by chromatography on a Histrap FF column (GE Healthcare, Chalfont St. Giles, UK). The fraction containing the recombinant H-NS protein was obtained by elution with 50 mM Tris-HCl buffer (pH8.3) containing 0.5 M imidazole, 50 mM NaCl, and 10% glycerol. The purity of the recombinant proteins was estimated by SDS-PAGE to be greater than 95%. Purification of H-NS_1-59_, H-NS_60-137_, H-NS_60-90_, H-NS_60-137_-AviTag, H-NS_60-90_-AviTag, and H-NS_91-137_-AviTag protein fragments with C-terminal HisTag relied on the same method.

### Preparation of Fluorescent Probes

Purified proteins (H-NS, H-NS_1-59_, H-NS_60-137_, H-NS_60-90_, H-NS_60-90_-AviTag) and the biotinylated random peptide were conjugated with Cy3 fluorescent dye according to manufacturer’s instructions (GE Healthcare). To prepare peptide tetramers, one µM streptavidin-Cy3 (Invitrogen, Carlsbad, CA) and 20 µM biotinylated peptide were mixed in 0.1 M Tris-HCl buffer (pH8.0) and incubated for 1 hour. To prepare streptavidin-Cy3 bound to biotin, which was used as a negative control, one µM streptavidin-Cy3 and 20 µM biotin were mixed in 0.1 M Tris-HCl buffer (pH8.0) and incubated for 1 hour.

### Kinetic Analysis of H-NS_60-90_-Cy3 and H-NS_60-90_ Tetramer-Cy3 Binding to Amosite

H-NS_60-90_ tetramer was prepared by mixing 1 µM streptavidin-Cy3 and 20 µM H-NS_60-90_-AviTag in 0.1 M Tris-HCl buffer (pH8.0). After incubation for 1 hour, unconjugated H-NS_60-90_-AviTag was removed using the centrifugal filter device BIOMAX-50K (Millipore, Billerica, MA). H-NS_60-90_-AviTag-Cy3 and its tetramer were diluted to the indicated concentrations in 0.5 ml of assay buffer [0.3 M Phosphate buffer (pH8.0) containing 0.3 M NaCl and 0.5% Tween 80]. Each dilution was then mixed with 0.05 mg amosite. After 10 min incubation at room temperature with rotary mixing, the amosite was precipitated by centrifugation at 20,000*g* for 5 min. The precipitated amosite was washed three times with 0.5 ml of assay buffer. Proteins bound to amosite were eluted by heating at 95°C for 5 min in 0.5 ml of elution buffer [0.1 M Tris-HCl buffer (pH8.0) containing 1% SDS]. Amosite fibers were precipitated by centrifugation at 20,000*g* for 10 min and examined under fluorescence microscope (Olympus BX60, Tokyo, Japan) to confirm complete elution of the fluorescent probe. The fluorescence intensity of supernatant was measured to determine the amount of Cy3-labeled protein bound to the amosite. Spectrofluorimeter FP-6500 (JASCO, Tokyo, Japan) was used to perform fluorescence measurements with the excitation and emission wavelengths set to 550 and 570 nm respectively. Probe binding data in the saturation binding curves were transformed to Scatchard plots to obtain the dissociation constants.

Since strictly molarity-based molecular affinity cannot be calculated for binding to inorganic surfaces, our measurements of dissociation constants rely on the following interpretation of molarity in relation to surface adsorption phenomena. If amosite asbestos were a biomolecule that interacted with the probe through a single binding site, the dissociation constant would be defined K_d_=[P_free_[[A_free_]/[C] where [P_free_], [A_free_] and [C] represent molar concentrations of the free probe, free asbestos and the bound probe-asbestos complex, respectively. Assuming a constant number of binding sites per unit of asbestos, its molarity can instead be expressed as the molar concentration of the binding sites in the suspension of asbestos fibers. [C] and [A_free_] in the original K_d_ equation would therefore refer to the respective molar concentrations of occupied and unoccupied (free) binding sites. By measuring the concentration of the bound probe [P_bound_] after its elution from asbestos surface, we can obtain both the molar concentration of the occupied binding sites on the asbestos surface ([C]=[P_bound_]), and the concentration of the free probe ([P_free_]= [P_initial_]-[P_bound_], where [P_initial_] represents the initial concentration of the probe added to asbestos suspension). To obtain the molar concentration of unoccupied binding sites [A_free_], we obviously need to know [A], the total molar concentration of the binding sites in the asbestos suspension. In its turn, [A] equals the maximum adsorption of the probe at the point of saturation, [P_max_], which can be estimated from the saturation binding curve. For all the [P_bound_] values below the saturation point [P_max_], the corresponding molar concentration of unoccupied binding sites [A_free_]=[A]-[C]=[P_max_]-[P_bound_]. Therefore, dissociation constants for asbestos probes can be obtained using the formula K_d_ =([P_initial_]-[P_bound_])([P_max_]-[P_bound_])/[P_bound_].

### Comparison of Binding Specificity of Protein Probes

H-NS_60-137_ tetramer, H-NS_60-90_ tetramer, and H-NS_91-137_ tetramer were diluted to a concentration of 20 nM in 0.5 ml of assay buffer. Amosite (0.05 mg) or wollastonite (0.05 mg) were added to 0.5 ml of assay buffer, incubated for 10 min at room temperature with rotary mixing and precipitated by centrifugation at 20,000*g* for 5 min. The precipitated fibers were washed three times with 0.5 ml of assay buffer, precipitated by centrifugation at 20,000*g* for 5 min, and resuspended with 0.05 ml of assay buffer. The samples were observed under fluorescence microscope equipped with U-MNG filter. Images were captured using a DP70 cooled charge-coupled device camera (Olympus).

### Quantification of the peptide tetramers’ adsorption on amosite

Peptide tetramers’ affinity to amphibole asbestos was estimated and compared by measuring each tetramer’s adsorption on amosite. Peptide tetramers and negative controls (Streptavidin-Cy3 bound to biotin, and the random peptide in free and streptavidin-assembled form) were diluted to a concentration of 20 nM in 0.5 ml of assay buffer. Amosite (0.05 mg) was added to 0.5 ml of assay buffer, incubated for 10 min at room temperature with rotary mixing and precipitated by centrifugation at 20,000*g* for 5 min. The precipitated amosite was washed three times with 0.5 ml of assay buffer. Peptide tetramers and negative controls bound to amosite were eluted by heating at 95°C for 5 min in 0.5 ml of elution buffer [0.1 M Tris-HCl buffer (pH8.0) containing 1% SDS] and the amosite was precipitated by centrifugation at 20,000*g* for 10 min. The fluorescence intensity of supernatant was measured to determine the amount of Cy3-labeled probe bound to the amosite. Spectrofluorimeter FP-6500 was used to perform fluorescence measurements with the excitation and emission wavelengths set to 550 and 570 nm respectively.

## Results

### Isolation of the H-NS fragment specific for amosite

**Figure 1:**
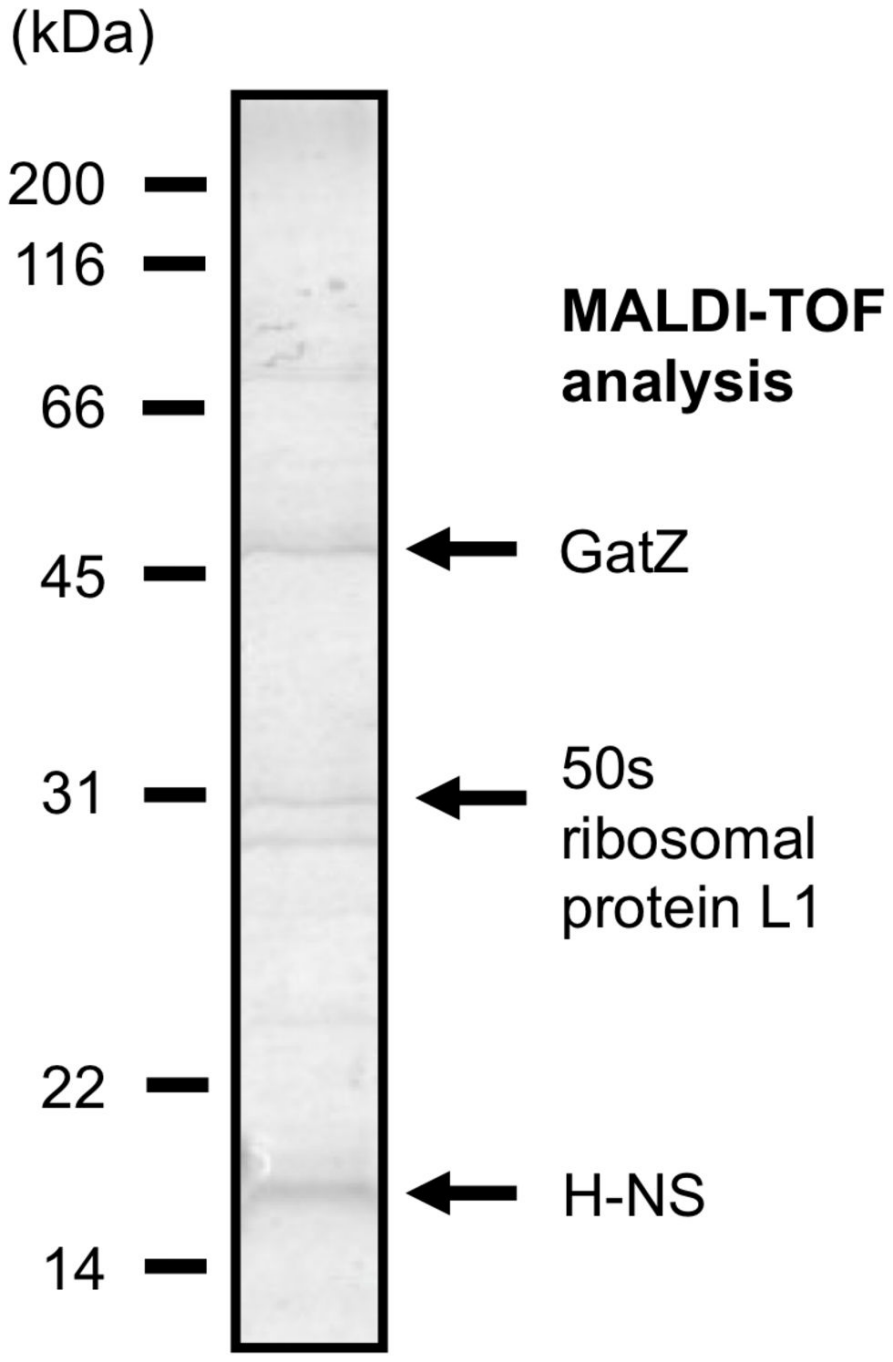
SDS–PAGE analysis of amosite-binding proteins isolated from *E. coli* lysate.

In our previous studies, we discovered several asbestos-binding proteins from bacterial lysate [2,3]. One of these proteins, *E. coli* adolase subunit GatZ, has already been used as an affinity probe for amphibole asbestos [3]. To improve the specificity of the amphibole probe, we conducted another screening for amphibole asbestos–binding proteins from *E. coli* lysate under more stringent conditions, and additionally identified H-NS protein using MALDI-TOF analysis (Figure 1). However, both GatZ and H-NS were found to also bind to wollastonite, an increasingly common asbestos substitute.

**Figure 2:**
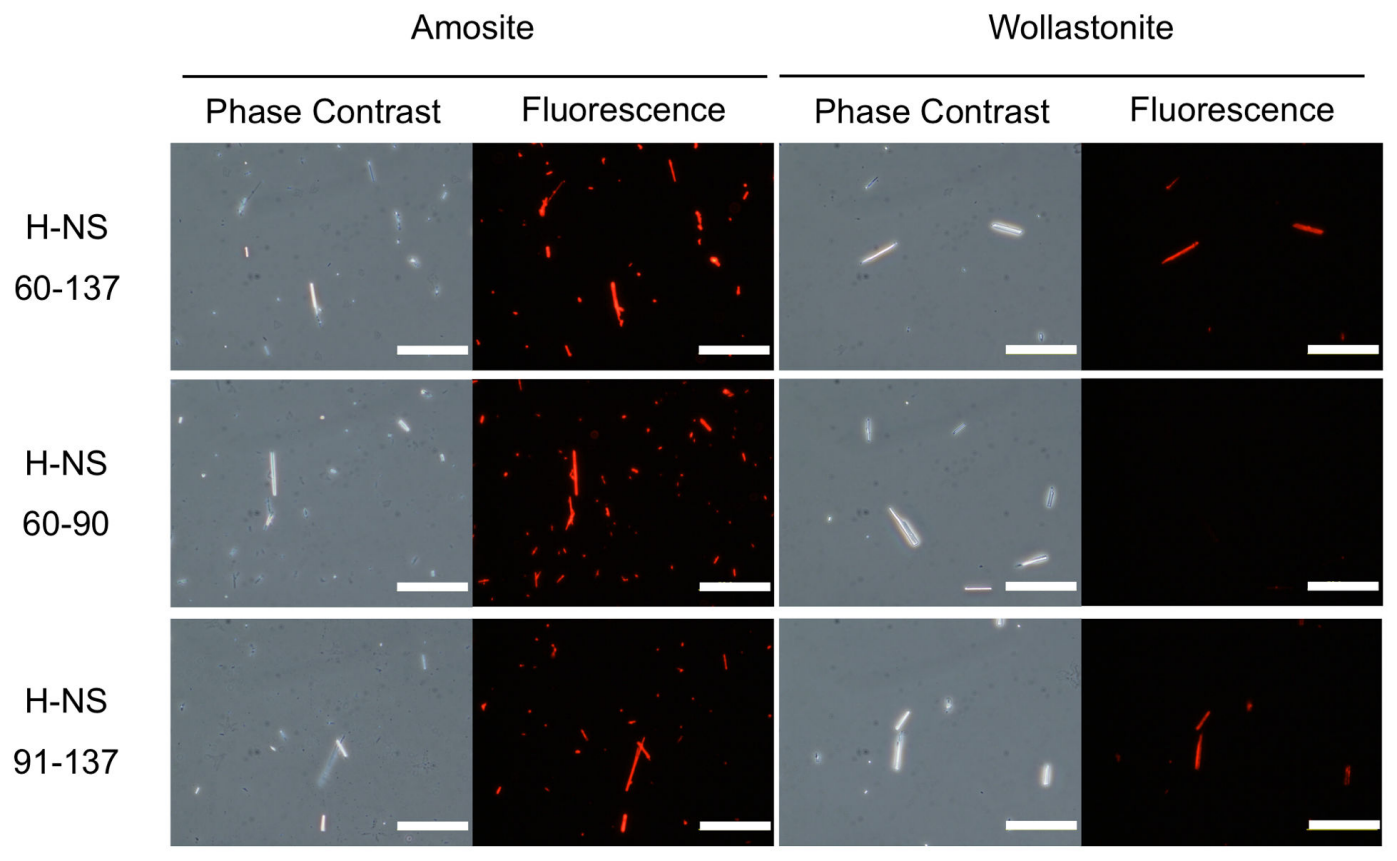
Specificity of fluorescently labeled tetramers of H-NS fragments. The fibers are stained with fluorescently labeled tetramers of the indicated H-NS fragments. Each pair of phase contrast and fluorescence micrographs of amosite and wollastonite fibers shows the same field of view. Bar, 50 µm.

A possible explanation for the insufficient specificity of amphibole-binding proteins is the presence of multiple binding sequences with different affinities and specificities. Therefore, in order to develop a more selective probe for amphibole asbestos, we attempted to isolate a protein fragment that is specific for amosite. GatZ is a globular protein, and has a largely hydrophobic core. Slicing this protein into several fragments would expose multiple hydrophobic residues, potentially changing the secondary structure and other properties of the asbestos-binding surface regions. We therefore decided to focus on H-NS, which comprises two distinct domains separated by a flexible linker at amino acids 60 to 89 [9,10]. We first sliced the H-NS into two fragments that largely correspond to N-terminal domain, H-NS_1-59_, and the flexible linker followed by C-terminal domain, H-NS_60-137_, and found that the former did not bind to either amosite or wollastonite, while the latter could bind to both kinds of fibers (data not shown). We further sliced H-NS_60-137_ into H-NS_60-90_ and H-NS_91-137_, and found that the former only bound to amosite, while the latter retained the affinity to both amosite and wollastonite (Figure 2). It seems likely that H-NS contains multiple amosite-binding sequences, all of which contribute to its high affinity to amosite asbestos. However, it may also contain wollastonite-binding sequence(s), and some of its amosite-binding sequences may be less specific (have affinity to wollastonite). Since H-NS_60-90_ did not bind to wollastonite, we concluded that it contained at least one binding sequence specific to amosite, and had no wollastonite-binding sequences.

### Increasing binding affinity of the probe by engineering streptavidin tetramer complex

All of the fragments of the H-NS protein had weaker affinity to amosite than H-NS itself, which made it difficult to evaluate the specificity of the individual fragments by fluorescence microscopy. The most likely explanation for the weaker binding of the protein fragments is that H-NS contains multiple low-affinity amosite-binding sequences or amino acids, all of which contribute to its affinity to amosite asbestos.

To improve the binding affinity of the individual protein fragments and, subsequently, the peptides carrying amosite-binding amino acid sequence, we engineered the probe to display multiple amosite-binding sequences on fluorescently labeled streptavidin. Streptavidin is a tetramer, with each of the four monomers carrying a binding site for biotin. Biotinylated protein fragments are thus multiply displayed on the streptavidin tetramer, and are oriented in the same plane and direction.

**Figure 3:**
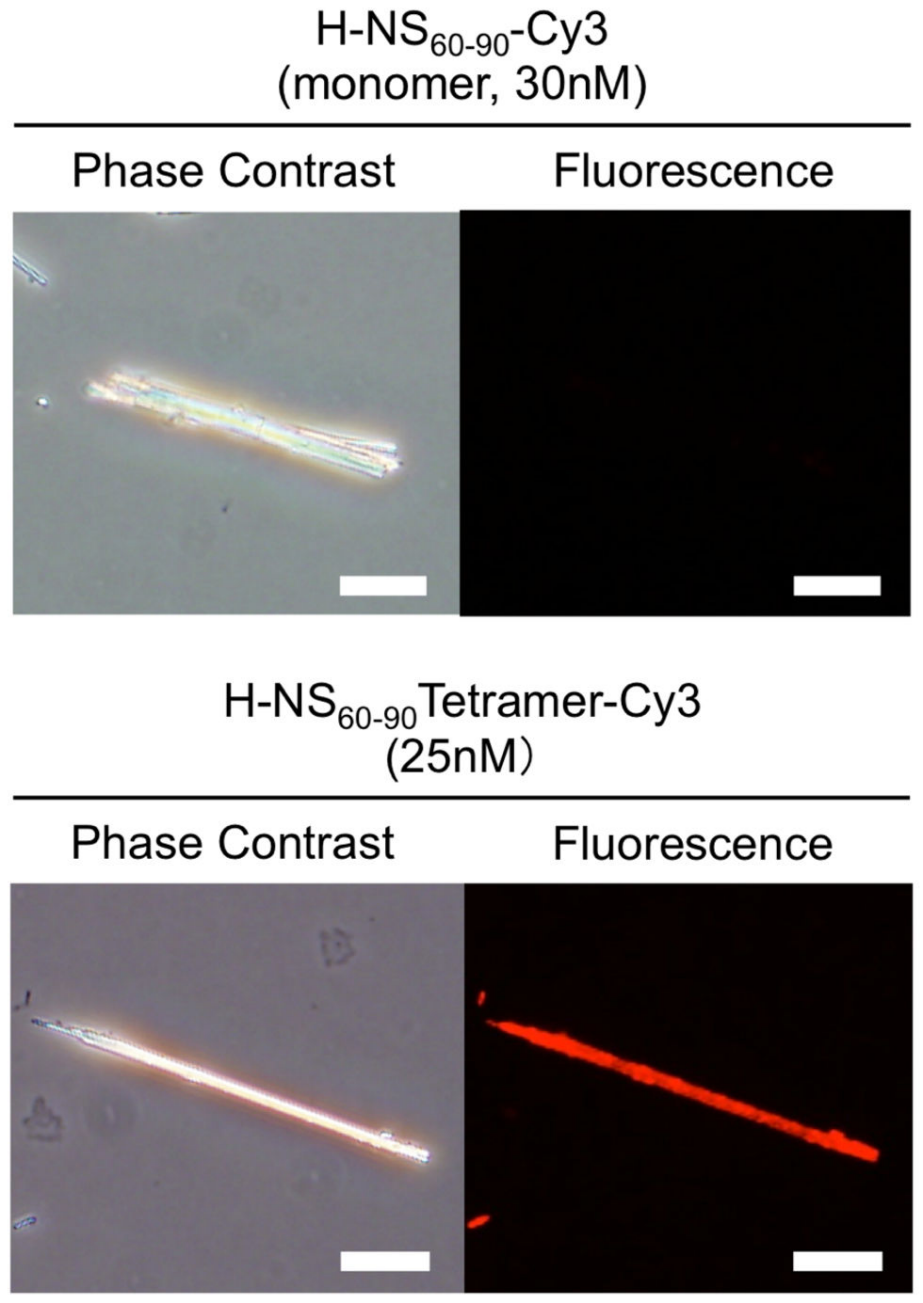
Amosite fibers stained with fluorescently labeled monomers and streptavidin-based tetramers of H-NS_60-90_. Each pair of phase contrast and fluorescence micrographs shows the same field of view. Due to lower affinity of H-NS_60-90_ monomer, its adsorption on amosite at the indicated concentration is minimal. Bar, 10 µm.

**Figure 4:**
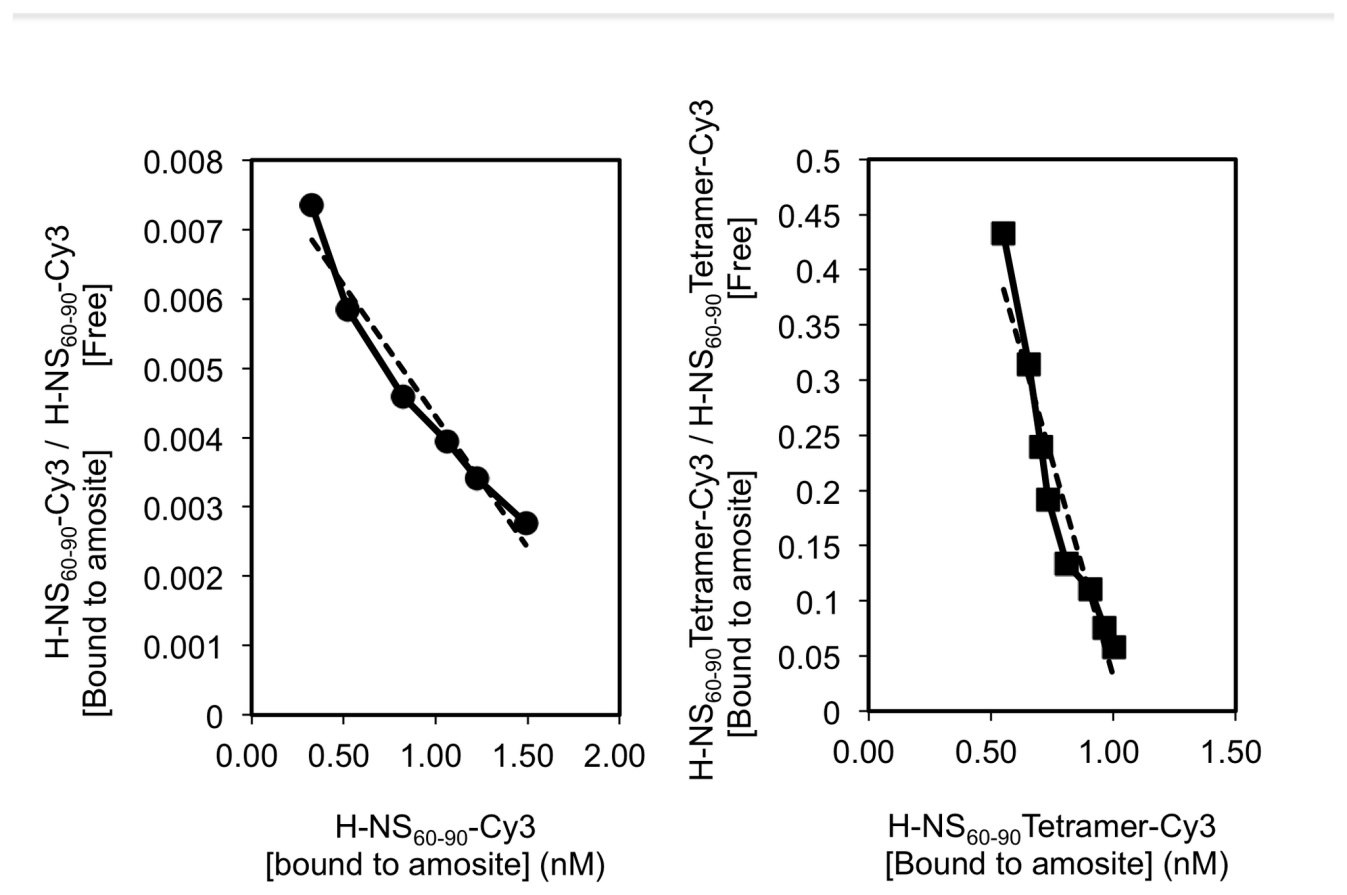
Scatchard analysis of H-NS_60-90_ monomer and its streptavidin-based tetramer’s adsorption on amosite.

Fluorescence microscopy of amosite fibers stained with the engineered streptavidin-based H-NS_60-90_ tetramer indicated a sizable improvement of the probe affinity to amosite (Fig. 3). We measured the magnitude of this improvement by calculating dissociation constants for the monomers and tetramers of the biotinylated H-NS_60-90_ fragments using Scatchard plots (Figure 4). Since this calculation required quantitative data on the probe adsorption, we initially attempted to directly measure fluorescence intensity of 0.05 mg amosite suspension following the binding and washing steps. Unfortunately, fluorescence measurements obtained by this method were not reproducible, possibly due to the light scattering by asbestos fibers. We therefore had to elute the bound probe from the asbestos surface, and measure the fluorescence of the eluate. Rather unexpectedly, complete elution of the bound tetramer probe from the surface of asbestos fibers required very harsh heat treatment of the fibers in 1% SDS buffer (5 min at 95°C). Milder elution methods (such as SDS treatment at lower temperatures or treatment with 6M guanidine hydrochloride solution) failed to achieve complete elution, suggesting extremely strong binding for at least some tetramers. The dissociation constant for the H-NS_60-90_ tetramer was 1.04 nM, which is approximately 250 times lower than that of H-NS_60-90_ monomer (263 nM) (Figure 4). To exclude the possibility that streptavidin itself binds to amosite or indirectly facilitates non-specific adsorption of peptides on amosite fibers, we also measured the binding affinity of a peptide composed of twenty different proteinogenic amino acids in random order (Bi-QMPEIVKFNHLWCRGDYSTA), both in the free and the streptavidin-assembled form, with an additional control of streptavidin bound to biotin. All of the control probes did not show measurable binding to amosite, which translates to K_d_ values of >1 µM.

A large increase in the affinity of the tetramer indicates the binding of at least two, and possibly all four H-NS_60-90_ fragments displayed on the streptavidin. After confirming the increase in affinity for H-NS_60-90_ tetramer, we constructed tetramers for other H-NS fragments and, subsequently, all the peptides used in this paper.

### Identification of the Binding Sequence

**Figure 5:**
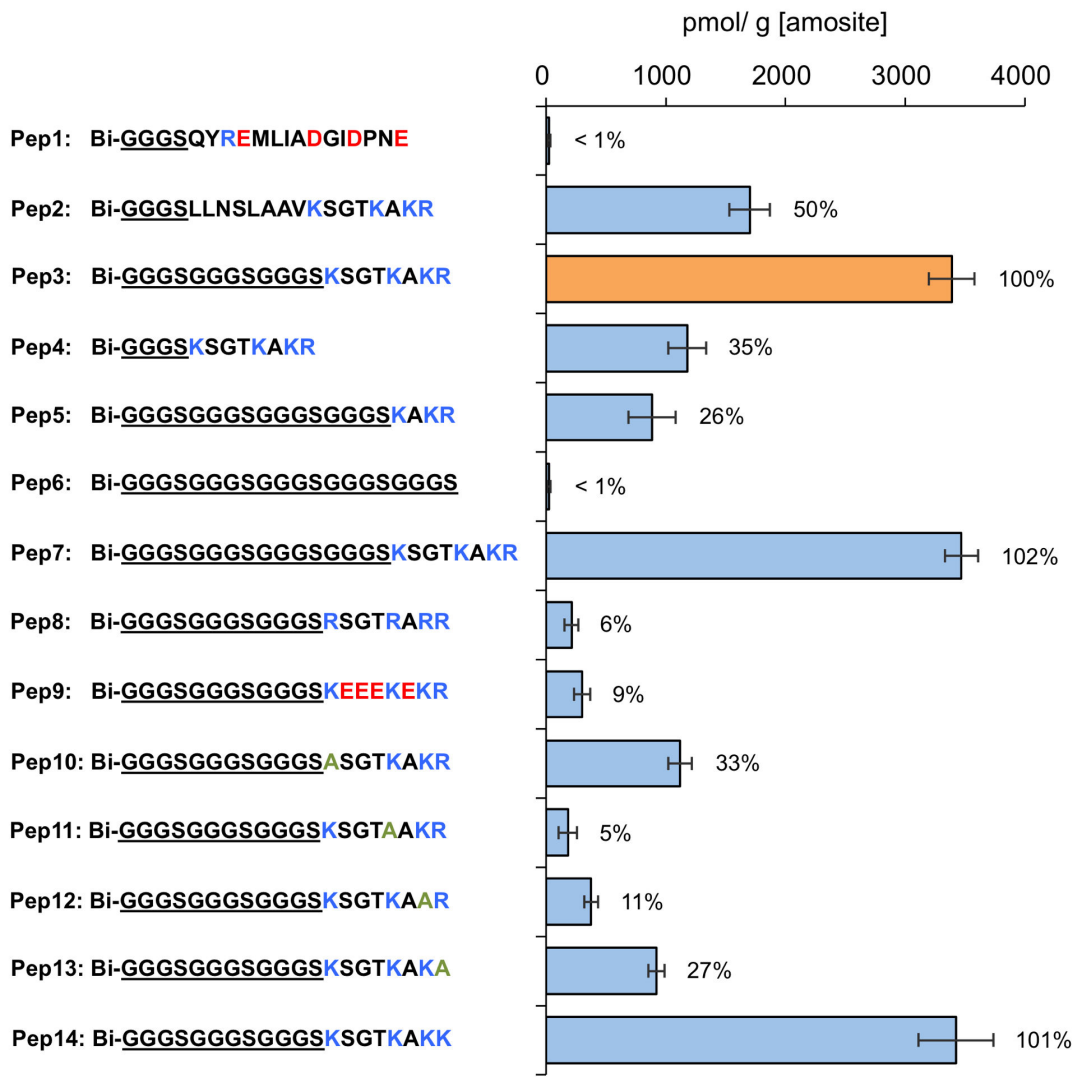
Adsorption of the engineered peptide tetramers on amosite. Pep1-Pep7 were used to identify the binding sequence and optimal linker length. Pep8-Pep14 were used to determine the binding mechanism and the contribution of individual amino acids of the binding sequence. The percentage values indicate the adsorption of each peptide tetramer relative to that of pep3, which is referred to as the “original binding sequence”.

A detailed examination of the amino acid sequence of H-NS_60-90_ protein fragment, which carries the amosite-binding sequence, reveals several biases in the distribution of charged and hydrophobic residues. The N-terminal half of this fragment (QYREMLIADGIDPNE) carries four negatively charged residues, several hydrophobic residues, and just a single positively charged arginine. On the other hand, the C-terminal half (LLNSLAAVKSGTKAKR) carries only positively charged residues near its own C-terminal, and many hydrophobic residues near its N-terminal. To identify the binding sequence, we constructed and tested two tetramers of peptides carrying N-terminal (pep1) and C-terminal (pep2) halves of H-NS_60-90_, connected to streptavidin by biotinylated GGGS linker. As shown in Figure 5, measurements of the probe adsorption on amosite indicated that the binding sequence was carried by the positively charged pep2. To find out whether the hydrophobic part of this peptide contributed to the amosite binding, we replaced it with the (GGGS)_2_ linker sequence to generate pep3 tetramer. Rather unexpectedly, the adsorption of pep3 was nearly double that of pep2 tetramer, suggesting only minimal contribution of hydrophobic residues. Nevertheless, removing the hydrophobic sequence altogether (pep4 tetramer) resulted in lower affinity than that of either pep2 or pep3. An attempt to shorten the binding sequence of pep3 by replacing its first four residues with GGGS (pep5 tetramer) also led to a drop in the affinity, confirming the optimal extent of the amosite-binding sequence (KSGTKAKR) in pep3. Finally, to ensure that the binding affinity of the flexible linkers we used does not bias our results, we also constructed the (GGGS)_5_ peptide tetramer (pep6) and found that its affinity for amosite was less than 1% of the original binding sequence in pep3. The most likely explanation for the differences in the affinity of pep2, pep3 and pep4 is the length of the linkers connecting the identified binding sequence to streptavidin. These linkers must have sufficient length and flexibility in order to enable all the binding sequences of the tetramer to access the surface of amosite fibers. The actual length of the linker in the binding buffer depends on both the number of amino acids and the secondary structure of the linker sequence. In pep2, the hydrophobic amino acid sequence (LLNSLAAV) adjacent to the amosite-binding domain (KSGTKAKR) essentially functioned as a part of the linker. This hydrophobic sequence is probably folded and is therefore stiffer and shorter than (GGGS)_2_, resulting in a weaker binding of the pep2 tetramer compared to that of pep3. Following this logic, the affinity of pep4 was the lowest among the three peptide tetramers due to insufficient length of its linker sequence.

While we did not establish the minimum sufficient length of the linker, extending its length by four amino acid residues (GGGS) in pep7 tetramer gave negligible improvement in terms of binding affinity relative to pep3, suggesting that the length of (GGGS)_3_ linker was sufficient to enable all the residues of the binding sequence to access the surface of amosite. Therefore, the peptides subsequently used to analyze the amosite-binding sequence of pep3 utilized the same (GGGS)_3_ linker, with the pep3 itself serving as a standard in comparing adsorption of different peptide tetramers on amosite (an indicator of binding affinity to amphibole asbestos).

The binding sequence identified here has several distinctive characteristics. Half of its amino acid residues (three lysines and the C-terminal arginine) carry positively charged side chains, while the remaining residues are mostly hydrophilic. The amino acid composition of the binding sequence seems to indicate a predominantly electrostatic interaction with negatively charged amosite fibers.

### Characterization of the binding sequence

One of the key questions in analyzing peptide interactions with inorganic surfaces is whether the binding affinity is primarily determined by the overall characteristics of the peptide, or by the presence of specific amino acids. For our binding sequence, this question boils down to estimating the relative importance of the overall surface charge (leading to electrostatic interaction between positively charged peptide tetramers and negatively charged amosite fibers) and the individual contribution of four positively charged amino acids, in particular that of three lysine residues.

To answer this question, we performed two major modifications of the original binding sequence. In an attempt to separate the contributions of overall positive charge and (also positively charged) lysine residues, one peptide had all of the lysines replaced with arginine residues (pep8), and in the other all four non-charged amino acids of the binding sequence were replaced with the negatively charged glutamic acid residues (pep9). Since both pep3 and pep8 carry four positively charged amino acids as well as a single negatively charged carboxyl on the C-terminal, the overall surface charge of the binding sequence should not be affected by the lysine to arginine substitution. If this overall charge were the main determinant of the peptide interaction with asbestos fibers, the affinity of the arginine-substituted pep8 should be similar to that of pep3. However, affinity of pep8 was only 6% relative to that of pep3, suggesting that the presence of lysine residues is more important that the overall surface charge of the peptide. It is even more notable that replacing all the non-charged residues with the negatively charged glutamic acids in pep9 was less detrimental than the lysine to arginine substitution, with pep9 tetramer retaining 9% of the affinity of pep3. While pep9 carries an equal number of positively and negatively charged amino acids, its overall charge at pH 8 should be negative due to the C-terminal carboxyl. However, pep9 did not completely lose its affinity to amosite, confirming that the presence of lysine side chains is the most important determinant of the peptide interaction with asbestos.

In order to estimate the relative importance of each positively charged residue in the original binding sequence (three lysine residues and one arginine), we separately replaced each of them with neutral alanine. As shown in Figure 5, all the resulting peptide tetramers had a much lower affinity to amosite as compared to pep3 carrying the original binding sequence. The magnitude of the reduction, however, was somewhat unexpected. For pep11 and pep12 tetramers, a loss of a single lysine residue resulted in dramatic changes in affinity, which dropped by 95% and 89%, respectively. On the other hand, affinity of pep10 and pep13 tetramers decreased by approximately 70% each, suggesting that the N- and C-terminal amino acids of the binding sequence are somewhat less important then the two closely spaced lysines inbetween.

Considering the low affinity of the arginine-substituted pep8, we assumed that the C-terminal arginine residue in the original binding sequence had a far smaller contribution to the binding than any of the three lysine residues. However, this assumption proved to be wrong: changing C-terminal arginine to alanine in pep13 tetramer resulted in noticeably weaker interaction with amosite, while replacing this residue with lysine in pep14 tetramer did not produce appreciable increase in the affinity. One possible explanation to this puzzle is the negative charge of the carboxyl on C-terminal residue. Rather than directly binding to the amosite surface, the side chain of C-terminal residue (whether lysine or arginine) may be “shielding” the carboxyl and preventing its detrimental interaction with negatively charged amosite asbestos or other lysine residues.

## Discussion

Several molecular engineering techniques have been combined to construct a peptide probe that has high affinity and specificity to the target inorganic material - amosite fibers, as well as four other kinds of asbestos fibers in the amphibole mineral group. Previously, we discovered a number of amosite-binding proteins by screening a bacterial lysate “library” of *E. coli* proteins. Here, we isolated a specific binding sequence from a less specific amosite-binding protein, and dramatically improved the affinity of the probe by displaying four binding sequences on fluorescently labeled streptavidin. The probable mechanism of the increase in affinity is by preventing dissociation of the probe after its initial contact (binding) with the asbestos surface. While a single peptide could rapidly dissociate after binding to asbestos, the dissociation of a streptavidin-based probe is probably much slower. Such a probe can be compared to a ship with four anchors. If its “anchor peptides” have affinity to asbestos, all or most of them could be expected to bind to asbestos surface following the initial contact. Streptavidin-based probe would dissociate from asbestos only if all of the “anchors” come loose at the same time, which is far less likely than the dissociation of a single peptide.

The developed probe has sufficient specificity to distinguish amphibole asbestos from wollastonite, natural fibrous silicate mineral that is widely recognized as an interfering mineral for asbestos testing. Polarized light microscopy (PLM), which is generally used to test bulk materials, requires a time-consuming multistep procedure to differentiate wollastonite from one type of amphibole asbestos, anthophyllite, as these materials share a number of optical characteristics [11]. Phase-contrast microscopy (PCM), commonly used for the airborne asbestos testing, is unable to distinguish asbestos from wollastonite [5,12]. Among the existing methods, only TEM provides the straightforward and reliable means to distinguish wollastonite from amphibole asbestos. In fact, precision of the TEM method has been evaluated using a mix of amosite and wollastonite fibers [13]. However, TEM is rarely used for asbestos monitoring due to high cost, time-consuming sample preparation and analysis, and in some cases limited availability [14].

In addition to the practical application of the developed probe to asbestos detection, some of our findings may have wider relevance to the research on the bioinorganic interfaces. First, the affinity of the discovered binding sequence to amosite fibers is probably due to electrostatic interaction between the positively charged side chains of lysine residues and negatively charged silanol groups on the amosite surface. Although the same binding mechanism could be expected to operate towards other negatively charged silicate fibers, such as wollastonite, the engineered probe had sufficient selectivity to distinguish amosite from wollastonite under fluorescence microscopy. In other words, highly selective binding could be achieved by seemingly indiscriminate means of electrostatic attraction. Second, a combination of high affinity and specificity for an inorganic material could be attained by displaying several low-affinity binding sequences. In our case, displaying just four low-affinity sequences with proper orientation resulted in drastic increase in affinity. Displaying multiple binding sequences on streptavidin is simpler and more flexible than the previously reported fusing of the binding peptides to subunits of multimeric proteins [15]. A similar approach could be fruitfully applied to shorter peptides identified using phage display method.

Since peptide binding to inorganic surfaces is still poorly understood, it is important to expand the arsenal of methods for discovery of binding sequences for different inorganic materials. The method described here, screening of the proteins in the bacterial lysate followed by identification of binding sequence, may lead to the selection of novel binding peptides for new as well as previously studied inorganic targets.

## Supporting Information

File S1 (DOC)
