# Supplementary material for: Molecular Engineering of a Fluorescent Bioprobe for Sensitive and Selective Detection of Amphibole Asbestos
Source: PLoS One. 2013 Sep 27;8(9):e76231. doi: 10.1371/journal.pone.0076231 (PMC3785465; doi:10.1371/journal.pone.0076231)
Supplement: File S1 — (DOC) [file pone.0076231.s001.doc]

Supporting information

DNA sequences encoding H-NS, H-NS_1-59_, H-NS_60-137_, H-NS_60-90_, H-NS_91-137_.

H-NS (Primer P1/P2)

ATGAGCGAAGCACTTAAAATTCTGAACAACATCCGTACTCTTCGTGCGCAGGCAAGAGAATGTACACTTGAAACGCTGGAAGAAATGCTGGAAAAATTAGAAGTTGTTGTTAACGAACGTCGCGAAGAAGAAAGCGCGGCTGCTGCTGAAGTTGAAGAGCGCACTCGTAAACTGCAGCAATATCGCGAAATGCTGATCGCTGACGGTATTGACCCGAACGAACTGCTGAATAGCCTTGCTGCCGTTAAATCTGGCACCAAAGCTAAACGTGCTCAGCGTCCGGCAAAATATAGCTACGTTGACGAAAACGGCGAAACTAAAACCTGGACTGGCCAAGGCCGTACTCCAGCTGTAATCAAAAAAGCAATGGATGAGCAAGGTAAATCCCTCGACGATTTCCTGATCAAGCAATAA

H-NS_1-59_ (Primer P1/P3)

ATGAGCGAAGCACTTAAAATTCTGAACAACATCCGTACTCTTCGTGCGCAGGCAAGAGAATGTACACTTGAAACGCTGGAAGAAATGCTGGAAAAATTAGAAGTTGTTGTTAACGAACGTCGCGAAGAAGAAAGCGCGGCTGCTGCTGAAGTTGAAGAGCGCACTCGTAAACTGCAG

H-NS_60-137_ (Primer P4/P2)

CAATATCGCGAAATGCTGATCGCTGACGGTATTGACCCGAACGAACTGCTGAATAGCCTTGCTGCCGTTAAATCTGGCACCAAAGCTAAACGTGCTCAGCGTCCGGCAAAATATAGCTACGTTGACGAAAACGGCGAAACTAAAACCTGGACTGGCCAAGGCCGTACTCCAGCTGTAATCAAAAAAGCAATGGATGAGCAAGGTAAATCCCTCGACGATTTCCTGATCAAGCAA

H-NS_60-90_ (Primer P4/P5)

CAATATCGCGAAATGCTGATCGCTGACGGTATTGACCCGAACGAACTGCTGAATAGCCTTGCTGCCGTTAAATCTGGCACCAAAGCTAAACGT

H-NS_91-137_ (Primer P6/P2)

GCTCAGCGTCCGGCAAAATATAGCTACGTTGACGAAAACGGCGAAACTAAAACCTGGACTGGCCAAGGCCGTACTCCAGCTGTAATCAAAAAAGCAATGGATGAGCAAGGTAAATCCCTCGACGATTTCCTGATCAAGCAA
